# Gastrointestinal Toxicity Prediction Not Influenced by Rectal Contour or Dose-Volume Histogram Definition

**DOI:** 10.1016/j.ijrobp.2023.07.002

**Published:** 2023-12-01

**Authors:** Douglas H. Brand, Sarah C. Brüningk, Anna Wilkins, Olivia Naismith, Annie Gao, Isabel Syndikus, David P. Dearnaley, Emma Hall, Nicholas van As, Alison C. Tree, Sarah Gulliford

**Affiliations:** ⁎ Division of Radiotherapy and Imaging, The Institute of Cancer Research, London, United Kingdom; † Department of Medical Physics and Biomedical Engineering, University College London, London, United Kingdom; ‡ Department of Health Science and Technology, ETH Zurich, Basel, Switzerland; § Swiss Institute for Bioinformatics (SIB), Lausanne, Switzerland; ║ Urology Unit; ¶ Radiotherapy Trials QA Group (RTTQA), Royal Marsden NHS Foundation Trust, London, United Kingdom; # Radiotherapy Department, Clatterbridge Cancer Centre, Liverpool, United Kingdom; ⁎⁎ Clinical Trials and Statistics Unit, The Institute of Cancer Research, London, United Kingdom; †† Department of Radiotherapy Physics, University College London Hospitals NHS Foundation Trust, London, United Kingdom

## Abstract

**Purpose:**

Rectal dose delivered during prostate radiation therapy is associated with gastrointestinal toxicity. Treatment plans are commonly optimized using rectal dose-volume constraints, often whole-rectum relative-volumes (%). We investigated whether improved rectal contouring, use of absolute-volumes (cc), or rectal truncation might improve toxicity prediction.

**Methods and Materials:**

Patients from the CHHiP trial (receiving 74 Gy/37 fractions [Fr] vs 60 Gy/20 Fr vs 57 Gy/19 Fr) were included if radiation therapy plans were available (2350/3216 patients), plus toxicity data for relevant analyses (2170/3216 patients). Whole solid rectum relative-volumes (%) dose-volume-histogram (DVH), as submitted by treating center (original contour), was assumed standard-of-care. Three investigational rectal DVHs were generated: (1) reviewed contour per CHHiP protocol; (2) original contour absolute volumes (cc); and (3) truncated original contour (2 versions; ±0 and ±2 cm from planning target volume [PTV]). Dose levels of interest (V30, 40, 50, 60, 70, 74 Gy) in 74 Gy arm were converted by equivalent-dose-in-2 Gy-Fr (EQD2_α/β= 3 Gy_) for 60 Gy/57 Gy arms. Bootstrapped logistic models predicting late toxicities (frequency G1+/G2+, bleeding G1+/G2+, proctitis G1+/G2+, sphincter control G1+, stricture/ulcer G1+) were compared by area-undercurve (AUC) between standard of care and the 3 investigational rectal definitions.

**Results:**

The alternative dose/volume parameters were compared with the original relative-volume (%) DVH of the whole rectal contour, itself fitted as a weak predictor of toxicity (AUC range, 0.57-0.65 across the 8 toxicity measures). There were no significant differences in toxicity prediction for: (1) original versus reviewed rectal contours (AUCs, 0.57-0.66; *P* = .21-.98); (2) relative- versus absolute-volumes (AUCs, 0.56-0.63; *P* = .07-.91); and (3) whole-rectum versus truncation at PTV ± 2 cm (AUCs, 0.57-0.65; *P* = .05-.99) or PTV ± 0 cm (AUCs, 0.57-0.66; *P* = .27-.98).

**Conclusions:**

We used whole-rectum relative-volume DVH, submitted by the treating center, as the standard-of-care dosimetric predictor for rectal toxicity. There were no statistically significant differences in prediction performance when using central rectal contour review, with the use of absolute-volume dosimetry, or with rectal truncation relative to PTV. Whole-rectum relative-volumes were not improved upon for toxicity prediction and should remain standard-of-care.

## Introduction

External beam radiation therapy (EBRT) is an internationally accepted treatment option for localized prostate cancer.^1^^,^^2^ In delivering such EBRT, a balance exists between achieving adequate prostate dose for tumor control while minimizing dose to the surrounding organs at risk (OARs), such as the rectum. A dose-response relationship between rectal dose and gastrointestinal toxicity is well established, leading to longstanding usage of rectal dose-volume constraints during treatment planning.^3^, ^4^, ^5^ Such constraints are often derived from a relative dose-volume histogram (DVH; ie, % of organ).^4^^,^^5^

Using dose-constraints in routine clinical practice requires delineation of the rectal contour in the same way as the constraint-defining study. However, interobserver contouring variability has been noted across many tumor sites and OARs.^6^ Interobserver rectal contouring variance can lead to differences of 10% to 20% in important relative-volume DVH parameters (eg, V50 Gy in 2 Gy/fraction [Fr]).^7^ Studies applying interobserver rectal dosimetry variations to prefitted rectal normal tissue complication probability (NTCP) models have shown either small^8^ or clinically significant^9^ differences in predicted toxicity. However, to our knowledge, no group has reported the implications of differing contouring methods on direct toxicity prediction using data from a large prospective quality-assured clinical trial.

**Fig. 1:**
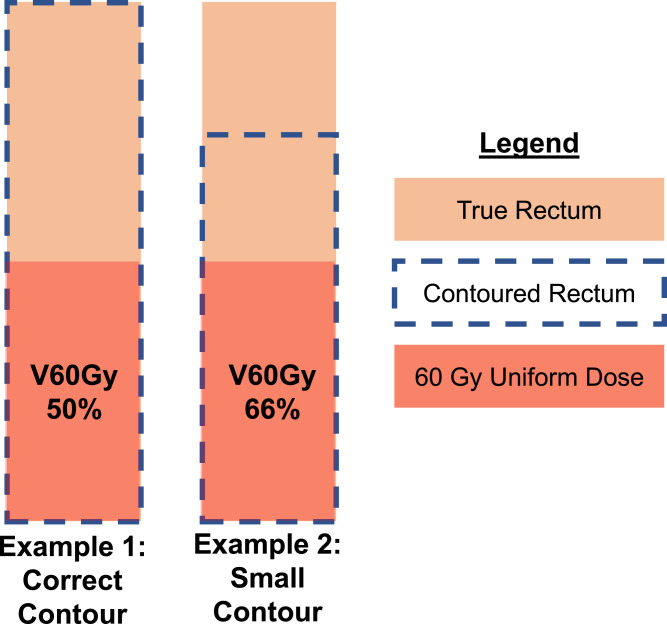
Demonstration of superior rectal border influencing relative dose-volume parameters. The rectal volume is contoured correctly in Example 1 but has a low superior border in Example 2. It can be seen that the smaller rectal volume of Example 2 results in an apparent increased relative V60 Gy(%) from 50% to 66%.

As an open-ended structure, observers may vary in how superiorly/inferiorly they define the limits of the rectal contour. For the superior border, this often occurs at distance from the planning target volume (PTV), thus increasing the nonirradiated rectal volume. This is particularly relevant for relative-volume DVH constraints, because the relative volume receiving, for example, 60 Gy (V60 Gy[%]) as a percentage will decrease if more nonirradiated rectum is contoured. Figure 1 demonstrates the potential issue with contouring variation at the superior border. We considered 3 methods that might be used to mitigate this effect.

First, interobserver variation in rectal contouring might be reduced by peer review and editing of volumes, aiming to ensure consistency with the trial protocol. For some studies reporting rectal dosimetric relationships, the rectum is recontoured to protocol definition before analysis;^5^^,^^10^ however, such review work requires considerable additional workload and is not universally undertaken.^11^^,^^12^ Routine prospective quality assurance (QA) work of 100 patients within the CHHiP trial demonstrated changes needed to meet protocol rectal definition for 43% of superior rectal borders and 32% of inferior borders.^13^ This rate of nonadherence suggests potential interest in reviewing contouring practice at a whole trial level. Whether rectal contour review is beneficial to direct toxicity prediction has, to our knowledge, not previously been reported.

Second, absolute volume (ie, cc of organ) DVH metrics could be utilized, as proposed since at least 2002.^14^ An attraction is that contouring more/less nonirradiated rectum will not alter the absolute volume receiving 60 Gy (V60 Gy[cc]). Multiple groups have compared relative and absolute rectal constraints, with variation in conclusion as to whether absolute^14^ or relative^15^, ^16^, ^17^ volumes are better for toxicity prediction. These studies included up to 331 patients and varied in conformality of radiation therapy. A larger study in the modern intensity modulated radiation therapy era may help to determine the optimal choice of relative or absolute volumes in toxicity prediction.

Third, variation in the superior/inferior extent of the contoured rectum might be reduced through procedural truncation relative to the PTV. Such truncation has previously been investigated, without a clear link to toxicity,^18^ although in a comparably small cohort of 23 patients. A larger study might therefore be warranted to see if the greater rectal consistency from PTV-based truncation might lead to improved toxicity prediction performance.

In summary, we define standard of care to be relative volume DVH dose-constraints generated from a rectum contoured by the original treating center clinician. This study will examine whether rectal toxicity prediction can be improved by any of 3 alterations to this definition: (1) contour review, (2) absolute volume DVH data, or (3) PTV-based rectal truncation. To answer these questions, we utilize data from a large phase 3 trial of conventional versus hypofractionated EBRT for localized prostate cancer (CHHiP).^19^

## Methods and Materials

### Trial and patient inclusion

The CHHiP study (ISRCTN97182923), clinical trial number ISRCTN97182923, has previously been reported in detail.^19^ Patients were eligible for inclusion in this substudy if: (1) a full protocol regimen was delivered (74 Gy in 37 Fr/60 Gy in 20 Fr/57 Gy in 19 Fr) and (2) radiation therapy treatment plans adequate for the recontouring process were available (ie, computed tomography [CT], dose, structures). Patients were excluded if either the original and/or reviewed rectal contour (defined in the following sections) were unavailable for analysis. Patients without toxicity data, or with baseline toxicity G1+, were excluded from toxicity analyses, per methodology described previously.^20^

### Contour review process

CHHiP defined the rectum as a solid structure “from the anus (usually at the level of the ischial tuberosities or 1cm below the lower margin of the PTV whichever is more inferior) to the recto-sigmoid junction. The rectosigmoid junction can usually be identified on the CT slice where the bowel turns anteriorly and to the left. This will give a length of 10-12cm in most cases.” In preparation for retrospective dosimetric analyses from the trial, the rectum contour (original rectum) was reviewed and recontoured where necessary to meet protocol definition (reviewed rectum) at the trial coordinating center, as previously reported.^20^ Briefly, 1 of 5 trained observers used VODCA (version 5.4.1; MSS Medical Software Solutions GmbH, Hagendorn, Switzerland) to open the patient treatment plan, converting to Digital Imaging and Communications in Medicine (DICOM) format where necessary. The rectal contour was reviewed and edited where necessary to meet the trial rectum definition, with particular attention paid to the superior and inferior borders. All DICOMs were then converted into CERR (Computational Environment for Radiotherapy Research,^21^ GitHub commits up to October 6, 2020) planC files and the relative volume DVH was recalculated for both original rectum and reviewed rectum, to ensure the same algorithm was used. For the original rectum, an absolute volume DVH was also calculated. For 8 patients, the original rectum was inadvertently overwritten during the review process, meaning they had to be excluded from this substudy.

### Procedurally generated truncated rectum

Using the planC files and scripts written in MATLAB (Mathworks, v2020a), the original rectum was copied and truncated so that the inferior and superior slices agreed with the PTV (rectum PTV ± 0 cm). This process was repeated with truncation at 2-cm superior and inferior to the superior-inferior extent of the PTV (rectum PTV ± 2 cm). For each of the new truncated rectum structures, the relative volume DVH was calculated in CERR. The choice of truncations to take forward to toxicity analysis (0 and 2 cm) was made pragmatically based on prior work in the field.^18^

### Statistical analyses

#### Rectal contour morphology comparison

A morphologic comparison was undertaken for reviewed versus original rectums, across a number of metrics. The cranio-caudal rectal length was calculated based on superior and inferior slice separation. Organ volume was taken from the DVH. The difference between original versus reviewed rectal lengths and volumes were then calculated. This was repeated for both PTV-based truncations. For each patient, the Dice similarity coefficient (DSC) was calculated for original versus reviewed rectum.^22^ For DSC display, graphical representation was by Tukey box-and-whisker plots.

#### Rectal dosimetric comparisons

For all DVHs, an equivalent dose in 2 Gy Fr (EQD2) correction was applied, with an α/β = 3 Gy chosen to represent late rectal toxicity.^20^^,^^23^^,^^24^ The dose-levels of interest were chosen based on the 74 Gy in 37 Fr regimen, to provide a spread of data across the DVH. These were V30, V40, V50, V60, V70, and V74 Gy. Dose levels for other fractionations were chosen to be EQD2 equivalent to these dose levels. The dose level conversions are summarized in Table E1. The dose levels of interest, where they refer to all 3 regimens corrected for EQD2, are starred: V30*, V40*, V50*, V60*, V70*, and V74* Gy. Volumes (% for relative DVHs, cc for absolute DVHs) at each dose-level were extracted from each patient's rectal DVHs. Graphical summarization of the DVH data was presented by mean averaging the volumes at each dose level.

The volume at each relative-volume DVH dose-level was plotted for original versus reviewed rectal contours. This was also done for relative and absolute volumes for the original rectum. At each dose-level, a comparison of the volumes was made by Wilcoxon signed-rank test.

### Toxicity comparison

#### Endpoints

Eight different unified rectal toxicities were considered: bleeding grade (G) ≥1, bleeding G2+, stool frequency G1+, stool frequency G2+, proctitis G1+, proctitis G2+, sphincter control G1+, and stricture/ulcer G1+. The amalgamation of these toxicity endpoints from the Radiation Therapy Oncology Group (RTOG), LENTSOM, and RMH scales has previously been described in detail.^20^ Patients with nonzero baseline toxicity were excluded for that endpoint. In short, events without intervention were deemed G1, and toxicity with intervention was G2. Any event recorded in the late follow-up assessments (6, 12, 18, 24, 36, 48, and/or 60 months) was sufficient for toxicity to be scored. To score as no toxicity, at least 50% (4/7) late follow-ups must have been completed. Toxicity endpoint frequencies for patients included in the toxicity analyses are shown in Table E2.

#### Original versus reviewed contours: Logistic model

The toxicity prediction values of the combined dose level data were estimated by a logistic model. Separately, for original and reviewed contours, all 7 relative-volume dose level bins (V30*, V40*, V50*, V60*, V65*, V70*, V74* Gy) were fitted simultaneously to a logistic regression model against each toxicity endpoint. Area under curves (AUCs) of the receiver operating curve for the whole logistic model were compared between original and reviewed contours by DeLong method.^25^ This process was repeated for 2000 bootstrap samples (sampled with replacement, stratified by toxicity) to provide AUC 95% CIs (2.5^th^-97.5^th^ bootstrap centiles) and estimates of test performance. Estimates of test performance for sensitivity, specificity, positive predictive value (PPV), and negative predictive value (NPV) were generated by the 632 bootstrapping method.^26^ This was preferred over 632+ because of quicker calculation and very low chance of near-perfect prediction. Estimates are termed for sensitivity632, specificity632, NPV632, and PPV632.

#### Relative volumes versus absolute volumes

Logistic models were fitted for each toxicity endpoint with the corresponding 7 dose bin values for both relative and absolute original rectal contours. This was bootstrapped and analyzed in the same manner as described previously for the original versus reviewed logistic model analysis. Comparison between the AUC for relative volumes and absolute volumes was by DeLong method.

### Truncated rectum analyses

The same approach was taken for truncated analyses with relative DVH data, except having 2 comparisons: original whole rectum versus 2 truncated original rectums, respectively PTV ± 0 cm and PTV ± 2 cm.

### Sensitivity analyses

Combining data from 3 different arms using EQD2 methodology naturally requires assumptions around dose-fractionation responses. We therefore performed sensitivity analyses where the toxicity modeling was repeated but restricted in turn to patients from each of the 3 arms.

### Significance levels

Because of multiple testing, corrections were applied for the interpretation of significance levels for *P* values. The most important significance tests were the 8 logistic model comparisons for each of the 4 key hypotheses (original vs reviewed; absolute vs relative volumes; whole rectum vs PTV ± 0 cm; whole rectum vs PTV ± 2 cm). These are interpreted as significant at the .0015 level by Bonferroni correction (32 tests). The dosimetric comparisons (volumes at each DVH dose level of interest) were considered exploratory in nature and interpreted at the .0001 significance level.

## Results

### Patient population

**Fig. 2:**
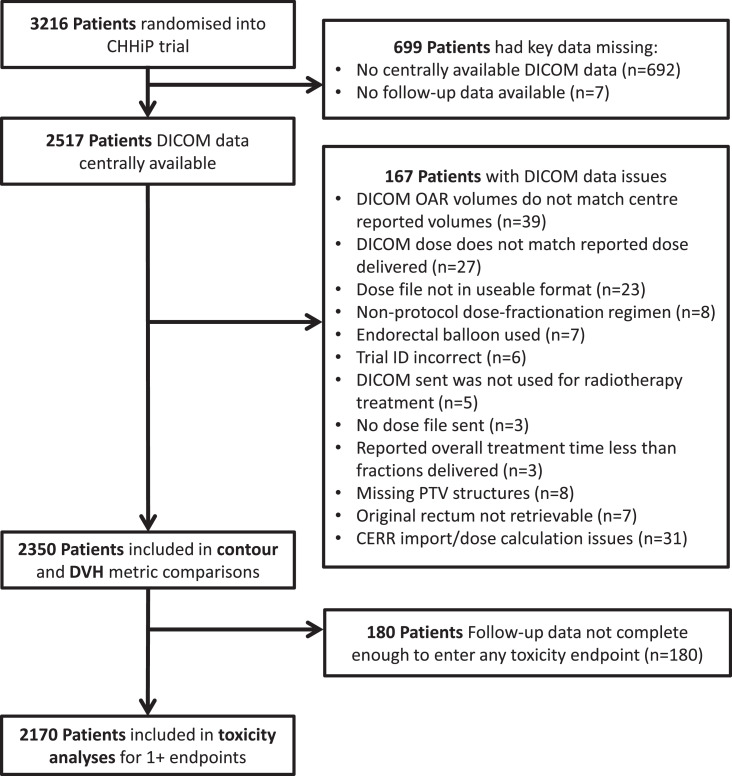
CONSORT-style diagram of substudy patient inclusion. *Abbreviations:* CERR = Computational Environment for Radiotherapy Research; DICOM = Digital Imaging and Communications in Medicine; DVH = dose-volume histogram; OAR = organ at risk.

From the CHHiP trial, 2350/3216 of randomized patients were included in 1 or more parts of this analysis. Figure 2 is a CONSORT-style flowchart indicating reasons for patient exclusion. The included patient cohort has similar baseline characteristics to the trial as a whole (Table E3).

### Contour morphology

Differences in the rectal length and volume between original and reviewed contours are shown in Table 1, both for whole rectum and both PTV-based truncations. As would be expected, truncation closer to the PTV reduces differences between original and reviewed rectal lengths and volumes. For whole rectum, the median length increased (+0.5 cm) while median volume decreased (–0.7 cm^3^). An example case demonstrating increased length with reduced volume is shown in Fig. E1. Increasing DSC agreement between original and review observer was also seen with truncation closer to the PTV (Fig. E2).

**Table 1:** Rectal lengths and volumes

	Rectal lengths (n = 2350)						Rectal volumes (n = 2350)					
	Original		Reviewed		Difference (reviewed minus original)		Original		Reviewed		Difference (reviewed minus original)	
Rectumdefinition	Med. (cm)	IQR (cm)	Med. (cm)	IQR (cm)	Med. (cm)	IQR (cm)	Med. (cm^3^)	IQR (cm^3^)	Med. (cm^3^)	IQR (cm^3^)	Med. (cm^3^)	IQR (cm^3^)
Whole rectum	9.3	8.3–10.5	10.0	9.0–11.0	0.5	0.0–1.5	66.0	52.6–86.3	64.3	51.0-84.0	−0.7	−7.9 to 2.7
PTV ± 2 cm	9.0	8.1–10.0	9.5	8.8–10.3	0.3	0.0–1.0	64.8	51.5–84.3	62.8	50.1-81.4	−0.4	−6.8 to 2.1
PTV ± 0 cm	6.9	6.0–7.5	6.9	6.0–7.5	0.0	0.0–0.0	53.1	41.9–69.0	50.9	40.4-66.6	−0.2	−4.8 to 0.5

Comparison of the rectal lengths and volumes between original and reviewed rectal contours, shown for whole rectum and both truncations.

*Abbreviations:* IQR = interquartile range; Med. = median; PTV = planning target volume.

### Rectal d

For both relative and absolute volumes, the volumes at each dose-level of interest are summarized for the original whole rectum in Fig. E3. Tabular summary data of each dose level of interest for all rectal definitions is provided in Table E4. The original and reviewed rectal dosimetry are compared for relative-volumes in Fig. E4 panel A, with several dose levels being significantly different between original and reviewed contours. More dose levels had significant original versus reviewed absolute-volume dose level volume differences (panel B). However, it should be noted that these differences are significant by Wilcoxon signed-rank, which tests directionality, whereas magnitudes of difference were small.

### Toxicity prediction of original versus reviewed rectal contours

The fitted logistic models of both original and reviewed rectums, for each toxicity endpoint, are presented in Table 2. The absolute values of AUC are fairly low, ranging from 0.57 to 0.65. The lower 95% CI boundary for all AUCs is above 0.5, implying the models are all significant but weak predictors of toxicity. The estimates of sensitivity, specificity, PPV, and NPV are also modest. Differences seen between original and edited models are generally reciprocal trade-offs of sensitivity for specificity and vice versa (eg, proctitis G1+). Critically, no significant difference was seen in toxicity prediction when using the original versus reviewed contours.

**Table 2:** Rectal toxicity prediction: Original versus reviewed rectal volumes

		Original							Reviewed						
Toxicity endpoint	No.	AUC	AUC 95% CI	Sens 632	Spec 632	PPV 632	NPV 632	AUC		AUC 95% CI	Sens 632	Spec 632	PPV 632	NPV 632	AUC: Original vs reviewed *P* value
Frequency G1+	1986	0.57	0.54–0.60	0.46	0.66	0.45	0.67	0.57		0.54–0.60	0.47	0.64	0.44	0.66	.9179
Frequency G2+	1982	0.60	0.56–0.64	0.61	0.56	0.18	0.90	0.60		0.56–0.63	0.67	0.47	0.17	0.90	.5431
Bleeding G1+	1969	0.60	0.57–0.63	0.42	0.73	0.44	0.72	0.60		0.57–0.62	0.43	0.71	0.43	0.72	.8507
Bleeding G2+	1967	0.60	0.57–0.64	0.48	0.66	0.19	0.88	0.59		0.55–0.63	0.46	0.67	0.19	0.88	.2050
Proctitis G1+	2105	0.58	0.56–0.61	0.55	0.55	0.40	0.70	0.58		0.55–0.60	0.62	0.49	0.39	0.71	.2884
Proctitis G2+	2104	0.57	0.54–0.61	0.66	0.43	0.12	0.92	0.58		0.54–0.61	0.64	0.46	0.12	0.92	.9438
Sphincter control G1+	2154	0.61	0.57–0.65	0.67	0.48	0.14	0.92	0.61		0.57–0.65	0.56	0.60	0.15	0.92	.5949
Stricture/ulcer G1+	2161	0.65	0.59-0.71	0.65	0.57	0.05	0.98	0.66		0.60-0.71	0.77	0.46	0.05	0.98	.9758

Separately for original and reviewed rectal contours, logistic models fitted to whole rectum relative DVH (%) V30*, V40*, V50*, V60*, V65*, V70*, and V74* Gy data for each endpoint. The Sens, Spec, PPV, and NPV are estimated by 632 method. The predictive ability (AUC) is compared between original and reviewed contours by DeLong comparison, with no significant differences seen for any toxicity endpoint.

*Abbreviations*: AUC = area under curve; DVH = dose-volume histogram; NPV = negative predictive value; PPV = positive predictive value; Sens = sensitivity; Spec = specificity.

### Toxicity prediction of relative versus absolute rectal volumes

The fitted logistic models of both relative (%) and absolute (cc) rectal dose-volume data, for each toxicity endpoint, are presented in Table 3. All models have lower 95% CIs above 0.5, with absolute values ranging from 0.56 to 0.63, implying significant but weak predictors. Although all relative volume models have either the same or better AUC estimate, no significant differences were seen between relative volume and absolute volume models across any toxicity endpoint.

**Table 3:** Rectal toxicity prediction: Relative versus absolute volumes

		Relative volumes						Absolute volumes						
Toxicity endpoint	No.	AUC	AUC 95% CI	Sens 632	Spec 632	PPV 632	NPV 632	AUC	AUC 95% CI	Sens 632	Spec 632	PPV 632	NPV 632	AUC: Relative vs absolute *P* value
Frequency G1+	1986	0.57	0.54–0.60	0.46	0.66	0.45	0.67	0.56	0.54–0.59	0.54	0.57	0.43	0.67	.6305
Frequency G2+	1982	0.60	0.56–0.64	0.61	0.56	0.18	0.90	0.57	0.53–0.61	0.34	0.74	0.18	0.88	.0675
Bleeding G1+	1969	0.60	0.57–0.63	0.42	0.73	0.44	0.72	0.59	0.56–0.62	0.56	0.57	0.39	0.73	.1299
Bleeding G2+	1967	0.60	0.57–0.64	0.48	0.66	0.19	0.88	0.59	0.55–0.62	0.49	0.62	0.18	0.88	.1719
Proctitis G1+	2105	0.58	0.56–0.61	0.55	0.55	0.40	0.70	0.57	0.55–0.60	0.60	0.51	0.40	0.70	.2808
Proctitis G2+	2104	0.57	0.54–0.61	0.66	0.43	0.12	0.92	0.57	0.53–0.61	0.67	0.43	0.13	0.92	.9126
Sphincter control G1+	2154	0.61	0.57–0.65	0.67	0.48	0.14	0.92	0.60	0.57–0.64	0.41	0.71	0.16	0.91	.6504
Stricture/ulcer G1+	2161	0.65	0.59-0.71	0.65	0.57	0.05	0.98	0.63	0.57-0.69	0.61	0.58	0.05	0.98	.2653

For original whole rectum contours, logistic models fitted, separately, to relative (%) and absolute (cc) DVH dose levels: V30*, V40*, V50*, V60*, V65*, V70*, and V74* for each endpoint. The Sens, Spec, PPV, and NPV are estimated by 632 method. The predictive ability (AUC) is compared between relative and absolute volume models by DeLong comparison, with no significant differences seen for any toxicity endpoint.

*Abbreviations*: AUC = area under curve; DVH = dose-volume histogram; NPV = negative predictive value; PPV = positive predictive value; Sens = sensitivity; Spec = specificity.

### Toxicity prediction of whole rectum versus truncated rectal definitions

The fitted logistic models for whole rectum versus the 2 truncated rectal definitions (PTV ± 2 and PTV ± 0 cm) are presented in Table 4. In the interest of space, only AUCs are presented, with full tables including sensitivity, specificity, PPV, and NPV shown in Tables E5 and E6. No significant differences in toxicity prediction (by AUC) are seen for any toxicity endpoint across both whole rectum versus PTV ± 2 cm and whole rectum versus PTV ± 0 cm.

**Table 4:** Rectal toxicity prediction: Whole rectum versus truncated definitions

		Whole rectum		PTV ± 2 cm			PTV ± 0 cm		
Toxicity endpoint	No.	AUC	AUC 95% CI	AUC	AUC 95% CI	Whole rectum vs PTV ± 2 cm *P* value	AUC	AUC 95% CI	Whole rectum vs PTV ± 0 cm *P* value
Frequency G1+	1986	0.57	0.54–0.60	0.57	0.55–0.60	.2361	0.57	0.55–0.60	.3831
Frequency G2+	1982	0.60	0.56–0.64	0.60	0.56–0.64	.9898	0.59	0.55–0.63	.3569
Bleeding G1+	1969	0.60	0.57–0.63	0.60	0.58–0.63	.0512	0.60	0.58–0.63	.4947
Bleeding G2+	1967	0.60	0.57–0.64	0.60	0.57–0.64	.9854	0.60	0.57–0.64	.6724
Proctitis G1+	2105	0.58	0.56–0.61	0.58	0.56–0.61	.7567	0.58	0.56–0.61	.6827
Proctitis G2+	2104	0.57	0.54–0.61	0.57	0.54–0.61	.9585	0.57	0.53–0.61	.3075
Sphincter control G1+	2154	0.61	0.57–0.65	0.61	0.57–0.65	.1463	0.61	0.58–0.65	.2668
Stricture/ulcer G1+	2161	0.65	0.59–0.71	0.65	0.59–0.71	.5129	0.66	0.60–0.71	.9836

Logistic models fitted for whole rectum, PTV ± 2 cm and PTV ± 0 cm (relative [%] dose levels: V30*, V40*, V50*, V60*, V65*, V70*, and V74*), for each endpoint. The predictive ability (AUC) is compared between whole rectal contours and each of the 2 truncated rectum models by DeLong comparison, with no significant differences seen for any toxicity endpoint.

*Abbreviations*: AUC = area under curve; PTV = planning target volume.

### Sensitivity analyses

Each of the previously mentioned toxicity models were refitted, restricted in turn to only patients from a single arm (eg, 37 Fr only). All model AUC comparisons (total = 96) remained nonsignificant at the .0015 level used in the main analysis. Two model AUC comparisons had a *P* value < .05 and are reported in the interest of openness, although they will not be further interpreted given multiplicity of testing. First, the proctitis G1+ model in 37 Fr only (n = 680), the original rectum AUC (0.61; 95% CI, 0.56-0.65) versus edited rectum AUC (0.59; 95% CI, 0.55-0.63), DeLong *P* value = .035. Second, the stool frequency G2+ model in 19 Fr only (n = 679), the use of relative DVH volumes (AUC, 0.62; 95% CI, 0.56-0.69) versus absolute DVH volumes (AUC, 0.56; 95% CI, 0.49-0.63), DeLong *P* value = .048. In both cases, any theoretical improvements favor the existing standard of care.

## Discussion

### Summary of study findings

With data from the CHHiP trial, this paper addresses a number of questions surrounding the rectal OAR contour for prostate EBRT. First, does the central review of rectal contours from trials improve toxicity modeling? Although small but significant differences were seen in original versus reviewed rectal dosimetry, no significant difference was found in toxicity prediction by logistic models. Given the substantial time cost of central rectal contour review, this would suggest the process can be omitted before rectal dosimetric analysis of ongoing major prostate EBRT trials (eg, RTOG 0924 [NCT01368588], PIVOTAL-BOOST [ISRCTN80146950], PACE [ISRCTN17627211]). Given every case has been seen by 2 observers, it also suggests that averaged minor deviations from trial rectal OAR definition are unlikely to significantly contribute to subsequent toxicity.

Second, relative rectal DVH data (%) has been compared with absolute volume DVH data (cc). Likewise, no significant difference in toxicity prediction was seen between the 2 approaches. This suggests that centers need not consider switching their practice from relative to absolute volume dose-constraints (or vice versa).

Third, the use of truncated versions of the original rectum (based on PTV) were examined. Again, no difference in toxicity prediction was seen between whole rectum and PTV ± 2 cm or PTV ± 0 cm. Although a truncated rectal contour saves contouring time, in the absence of toxicity prediction improvement, retaining whole rectum as the standard definition would likely be sensible. Maintaining a consistent definition of the rectum (ie, whole rectum) is beneficial to the implementation of multicenter trials and to the external generalizability of dosimetric analysis from such trials. Additionally, for patients receiving noncoplanar radiation therapy, contouring the whole rectum is critical to prevent possible excess dose in parts of the rectum at distance from the PTV.

### Observer effects on rectal morphology and dosimetry

QA for the prostate dose-escalation Medical Research Council RT-01 trial had 13 observers contour 3 cases, with large interobserver differences (up to 7 cm) noted for the superior border of the rectum.^27^ This prompted a clearer definition of the rectal superior border (the rectosigmoid junction), which was carried over into the CHHiP trial. The results here show that differences of up to 7 cm in length are occasionally still seen between original and reviewed rectal lengths in this study, although in general differences were smaller (interquartile range ≈ –2 to 4 cm). This is likely the result of 2 observers disagreeing on the superior limit of the recto-sigmoid junction, a challenging point given the open rectal ended structure.

A similar QA process for the AIROPROS01-02 trial had 18 observers contour the rectum on 4 prostate cases, with a strict definition of the inferior and superior rectal limits.^7^ This definition perhaps drove the lower interobserver variability seen compared with the RT-01 QA study, with no observer >1 cm from the global mean for the cranial or caudal border. Despite tighter contouring consistency, they noted interobserver rectal cumulative DVH differences of 15% to 20% (over the range V40-V65 Gy). The original versus reviewed whole rectum relative volume DVH differences were generally smaller in this study (median <3%).

### Observer effects on rectal NTCP

Prefitted NTCP models have been used to interpret interobserver difference in rectal DVH dosimetry. Fiorino et al^8^ had 10 patients’ rectums contoured by 3 observers. Dosimetry was fed into a prefitted Lyman Kutcher-Burman NTCP model, assuming a dose of 75.6 Gy. As with AIROPROS01-02, interobserver differences resulted in some large DVH changes, for example, 10% to 12% in the V50 to V65 range. However, the standard deviation (SD) in NTCP75.6 probability was just 0.7%, suggesting limited predicted toxicity alteration. Roach et al^9^ reported 2 NTCP-modelled cohorts: first, 3 observers of 35 patients; second, 10 observers of 5 patients. Interobserver rectal NTCP differences were found: cohort 1 (SD, 1.2%) and cohort 2 (SD, 2.5%), implying that the least extreme 95% of observer variability will cover a 10% range of NTCP difference. Such magnitudes suggest observer variability might influence toxicity prediction. However, our study, using collected toxicity data rather than NTCP values, does not show that review of the rectum by a second observer altered toxicity prediction.

### Absolute volume dosimetry

Kupelian et al^14^ reported on 128 patients receiving EBRT (70-78 Gy in 2-2.5 Gy Fr), fitting both relative and absolute rectal volume multivariate models for late rectal bleeding. Absolute volume was an independent predictor of toxicity, whereas relative volume was not. In another study, Koper et al^28^ reported on 199 patients receiving 66 Gy (2 Gy per Fr) of EBRT, with a 33% rate of any rectal bleeding at 3 years. They fit Kaplan-Meier models for rectal bleeding for relative and absolute volumes, trying different “cut-off” dose values to create 2 groups, then comparing the log-rank *P* values found, without correction for multiple testing. They noted that for the solid rectum models, the relative volume outperformed the absolute volumes. Vargas et al^15^ reported in 2005 on 331 patients treated with EBRT (63-79.2 Gy, 1.8 Gy per Fr) in a prospective dose-escalation protocol, with a 10.3% rate of late rectal toxicity G2+. They found relationships between both relative and absolute rectal volumes with toxicity, inferring stronger relationship for relative volumes through lower model *P* values.

More recently, in 2018, Kotabe et al^16^ reported a small retrospective study of 82 patients receiving 76 Gy/38 Fr EBRT as primary radiation therapy, with low late rectal bleeding rate (3.2%). Despite examining late toxicity, dosimetry was EQD2 adjusted using an α:β ratio of 10 Gy. Relative and absolute rectal DVH parameters were fitted sequentially, identifying only relative V60 Gy as significant for rectal bleeding. Paleny et al^17^ retrospectively reported on 285 patients receiving various forms of EBRT as salvage, adjuvant, and primary radiation therapy for prostate cancer (60-78 Gy in 2 Gy Fr), with low rates of G2+ late radiation proctitis (3%). By univariate logistic regression, multiple relative dose-volume parameters were significantly related to G1+ late radiation proctitis but no absolute dose-volume parameter. This study is hampered by its retrospective nature, heterogenous patient groups, and low event rates of relevant toxicity (ie, G2+).

Overall, the data on this subject are highly heterogenous, with multiple retrospective and small studies, often with low event rates. Results for and against absolute volumes have been seen. The data in this paper provide a sample size larger than every preceding study combined, prospectively collected, with good follow-up duration (the 5-year follow-up data set) and reasonable toxicity event rates. For the solid rectum we find no evidence for the benefit of absolute volumes over relative volumes.

### PTV-based rectal truncation

Prior data examining truncation of rectal contours is limited. Retrospectively contouring 163 primary/salvage EBRT prostate patients, Nitsche et al^18^ examined 3 different rectal definitions (chosen from 13 based on DVH heterogeneity): RTOG definition, PTV ± 1 cm, PTV ± 0 cm. No relationship was seen between various DVH parameters and worst late rectal inflammation G1+ (Common Terminology Criteria for Adverse Events). We are not aware of other data examining differential truncation with regard to late toxicity, although it has been reported for acute toxicity.^29^ The data in this paper are far larger (n ∼ 2000 for all endpoints), with demonstrable significant relationships between dosimetry and toxicities (seen as AUCs; 95% CIs not encompassing 0.5 of no effect). The size of this study suggests we can be confident that PTV-truncation–based definitions for solid rectum do not result in better rectal toxicity prediction.

### Strengths of the study

The previously mentioned prior studies have generally suffered from at least 1 of a few common issues: small sample size, retrospective toxicity collection, or analysis of strength of association by magnitude of *P* values. This current study is very well powered, with ∼2000 patients in each toxicity model. This generates tight, unbiased, 95% CIs for model predictive AUCs and greatly diminishes the chance of type II error. Additionally, the toxicity data are prospectively collected, with standardized toxicity scales and good follow-up duration (5-year data set). The use of prospective toxicity data, with exclusive inclusion of those with zero baseline toxicity, are strengths of this study for the elucidation of dose-toxicity relationships.

### Limitations of the study

The use of 1 central reviewer per rectal contour is a limitation. There will always be marginal cases when interpreting the rectal border, so ideally one would have multiple observers to obtain a gold-standard Simultaneous Truth and Performance Level Estimation (STAPLE) contour for each rectum. However, the use of a >2000 patient cohort makes such an approach impractical, meaning this study instead relies upon the sample size reducing the effect of cases where substantial interobserver heterogeneity may exist. We do note that given these data arise from a clinical trial it is possible that contour review may be more effective in centers not participating in clinical trials, something we cannot examine within this study.

Regarding the rectal OAR structure, we have limited this study to consider the rectum as a solid structure rather than rectal wall, therefore findings may not be extrapolatable to centers using rectal wall or surface dose parameters. It is, of course, possible that such rectal wall or surface maps may offer improved toxicity prediction; however, that is outside of the scope of the current study. The rectal doses are planned rather than delivered doses, a necessity given the absence of daily soft tissue imaging. It is plausible that rectal dose-accumulation mapping based on delivered dose might find stronger dose-toxicity relationships. Additionally, structures beyond the rectum (eg, bowel) may produce gastrointestinal toxicity; however, such alternative structures were not available in this study.

The choices of toxicity endpoints are also limitations to this exercise. Grade 1 and 2 toxicity suffers from noisy signal in an elderly population who may develop symptoms in the absence of radiation. However, modeling more severe (G3+) toxicities is very difficult because of their rarity, generating very severe class imbalance.

A further consideration is that a relatively basic model has been selected (logistic regression without variable selection). It would be possible to undertake more complex modeling methodologies (ie, including clinical or genomic data to improve NTCP predictions^30^), but this decision was made to permit comparison of easily understood like-for-like models for the different rectal definitions.

Finally, this study would be optimally conducted by performing each question as a randomization within a randomized controlled trial to allow the treatment planning system to optimize based on the different rectal definitions. For obvious funding reasons this is unlikely to happen, meaning our post hoc analysis of a prospective trial is likely the best feasible approach.

### Recommendations for future research

Although current treatment planning systems generally infer rectal toxicity from dosimetry alone (via DVHs), multiomic models incorporating, for example, clinical and genomic information to produce an NTCP would likely better predict rectal toxicity.^30^ Incorporation of such multiomic models into treatment planning systems may challenge the primacy of the OAR in the future prediction of individual toxicity.

Another consideration is that after CHHiP, treatments have become even more conformal. For example, although CHHiP used PTV margins of up to 1 cm, the PACE-B trial (randomizing patients with low-intermediate risk prostate cancer between stereotactic body radiation therapy and conventional radiation therapy) used margins of 3 to 5 mm. Conclusions about the use of relative-volume DVHs in those receiving such conformal stereotactic body radiation therapy treatments may not hold because of the much lower quantity of irradiated rectum. Testing relative versus absolute volume rectal DVH parameters for toxicity prediction in those receiving stereotactic body radiation therapy would be of interest.

In the further future, developments in computer analysis may make the role of the OAR less clearly defined. Given a sufficiently large training data set, it is possible that a deep learning network may be able to infer the critical CT voxels for subsequent “rectal” toxicity without human delineation of the organs. This may allow incorporation of information from previously undefined contributing structures lying outside of the normal rectal OAR.^31^

## Conclusion

Using data from the CHHiP trial, this study has demonstrated that central rectal contour review confers no statistically significant improvement for the prediction of rectal toxicity. We have then demonstrated no statistically significant change in toxicity prediction from the use of absolute versus relative volume DVHs. Finally, we showed that PTV-based truncation of the rectum (at PTV ± 0 cm and PTV ± 2 cm) also failed to statistically significantly improve toxicity prediction.

To summarize, whole rectum relative DVHs appear to be suitable to remain as the general status quo for toxicity modeling. However, it is clear that dosimetry data alone, as used for DVH dose constraints, result in weak predictors. Efforts to combine dosimetry from whole rectum relative DVHs (potentially via dose-accumulation mapping) with other predictors (eg, clinical factors, genomic, baseline symptom data) should be considered for treatment planning system NTCP prediction.

## Appendix Supplementary materials

Appendix Image, application 1

## Footnotes

Supplementary material associated with this article can be found, in the online version, at doi:10.1016/j.ijrobp.2023.07.002.
