# Supplementary material for: Gastrointestinal Toxicity Prediction Not Influenced by Rectal Contour or Dose-Volume Histogram Definition
Source: Int J Radiat Oncol Biol Phys. 2023 Dec 1;117(5):1163–73. doi: 10.1016/j.ijrobp.2023.07.002 (PMC10680426; doi:10.1016/j.ijrobp.2023.07.002)
Supplement: Appendix [file mmc1.docx]

**Supplement**

**Contents**

[Supplementary Table 1. EQD2 Corrected Rectal Dose-Levels of Interest with Corresponding Physical Doses by Regimen 2](#_Toc142336280)

[Supplementary Table 2. Toxicity Endpoint Frequencies 3](#_Toc142336281)

[Supplementary Table 3.Treatment Arm and Disease Characteristic of Patients in this Substudy Compared to the CHHiP Trial as a Whole 4](#_Toc142336282)

[Supplementary Figure 1. Example of Whole Rectum Contour Review Causing Longer Rectal Length But Reduced Rectal Volume 5](#_Toc142336283)

[Supplementary Figure 2. Dice Similarity Coefficients Between Original and Reviewed Rectum for Whole and PTV-Truncated Rectum. 6](#_Toc142336284)

[Supplementary Figure 3. Volumes at each dose-level of interest for the original whole rectum, by trial arm. 7](#_Toc142336285)

[Supplementary Table 4. Volumes at each dose-level of interest for the original whole rectum, by trial arm. 8](#_Toc142336286)

[Supplementary Figure 4. Volumes at Each Dose Level of Interest 9](#_Toc142336287)

[Supplementary Table 5. Rectal Toxicity Prediction: Whole Rectum vs PTV±2cm 10](#_Toc142336288)

[Supplementary Table 6. Rectal Toxicity Prediction: Whole Rectum vs PTV±0cm 11](#_Toc142336289)

# Supplementary Table 1. EQD2 Corrected Rectal Dose-Levels of Interest with Corresponding Physical Doses by Regimen

The dose levels were chosen in the 74 Gy in 37 fraction regimen. These were then converted to EQD2. A dose level was then set in each other regimen to be equal in EQD2.

| **Dose Level**  **From 74 Gy in 37 Fraction Regimen** | **EQD2-Corrected Dose Level**  **α/β = 3 Gy** | **CHHiP Trial Arms** | | |
| --- | --- | --- | --- | --- |
|  |  | **74 Gy** | **60 Gy** | **57 Gy** |
|  |  | **Dose (Gy)** | **Dose (Gy)** | **Dose (Gy)** |
| V30* | 22.9 | 30 | 26.4 | 26.1 |
| V40* | 32.6 | 40 | 34.5 | 34.1 |
| V50* | 43.5 | 50 | 42.5 | 41.8 |
| V60* | 55.5 | 60 | 50.3 | 49.5 |
| V65* | 61.8 | 65 | 54.2 | 53.3 |
| V70* | 68.5 | 70 | 58.0 | 57.0 |
| V74* | 74.0 | 74 | 61.1 | 60.1 |

# Supplementary Table 2. Toxicity Endpoint Frequencies

Showing the baseline rates of toxicity for patients included in one or more toxicity analyses. Abbreviations: GX+ = Grade X or more.

| **Rectal Endpoints &**  **Grades of Interest** | **Dose-Fractionation Regimen** | | | | | | **Total** | |
| --- | --- | --- | --- | --- | --- | --- | --- | --- |
|  | **57 Gy in**  **19 fractions** | | **60 Gy in**  **20 fractions** | | **74 Gy in**  **37 fractions** | |  |  |
|  | **No.** | **%** | **No.** | **%** | **No.** | **%** | **No.** | **%** |
| **Bleeding G1+** | |  |  |  |  |  |  |  |
| No | 469 | 70.6% | 430 | 64.0% | 430 | 67.9% | **1,329** | **67.5%** |
| Yes | 195 | 29.4% | 242 | 36.0% | 203 | 32.1% | **640** | **32.5%** |
| Excluded | 71 |  | 65 |  | 65 |  | **201** |  |
| **Bleeding G2+** | |  |  |  |  |  |  |  |
| No | 578 | 87.0% | 565 | 84.1% | 542 | 85.9% | **1,685** | **85.7%** |
| Yes | 86 | 13.0% | 107 | 15.9% | 89 | 14.1% | **282** | **14.3%** |
| Excluded | 71 |  | 65 |  | 67 |  | **203** |  |
| **Frequency G1+** | |  |  |  |  |  |  |  |
| No | 430 | 63.2% | 420 | 62.4% | 384 | 60.7% | **1,234** | **62.1%** |
| Yes | 250 | 36.8% | 253 | 37.6% | 249 | 39.3% | **752** | **37.9%** |
| Excluded | 55 |  | 64 |  | 65 |  | **184** |  |
| **Frequency G2+** | |  |  |  |  |  |  |  |
| No | 599 | 88.2% | 577 | 86.0% | 537 | 85.0% | **1,713** | **86.4%** |
| Yes | 80 | 11.8% | 94 | 14.0% | 95 | 15.0% | **269** | **13.6%** |
| Excluded | 56 |  | 66 |  | 66 |  | **188** |  |
| **Proctitis G1+** | |  |  |  |  |  |  |  |
| No | 498 | 69.5% | 443 | 62.6% | 428 | 62.9% | **1,369** | **65.0%** |
| Yes | 219 | 30.5% | 265 | 37.4% | 252 | 37.1% | **736** | **35.0%** |
| Missing | 18 |  | 29 |  | 18 |  | **65** |  |
| **Proctitis G2+** | |  |  |  |  |  |  |  |
| No | 651 | 90.9% | 629 | 88.8% | 598 | 87.9% | **1,878** | **89.3%** |
| Yes | 65 | 9.1% | 79 | 11.2% | 82 | 12.1% | **226** | **10.7%** |
| Excluded | 19 |  | 29 |  | 18 |  | **66** |  |
| **Sphincter Control G1+** | | |  |  |  |  |  |  |
| No | 665 | 91.1% | 651 | 88.7% | 604 | 87.5% | **1,920** | **89.1%** |
| Yes | 65 | 8.9% | 83 | 11.3% | 86 | 12.5% | **234** | **10.9%** |
| Excluded | 5 |  | 3 |  | 8 |  | **16** |  |
| **Stricture/Ulcer G1+** | | |  |  |  |  |  |  |
| No | 716 | 97.5% | 704 | 95.8% | 663 | 95.8% | **2,083** | **96.4%** |
| Yes | 18 | 2.5% | 31 | 4.2% | 29 | 4.2% | **78** | **3.6%** |
| Excluded | 1 |  | 2 |  | 6 |  | **9** |  |
| **Total** | **735** | **100%** | **737** | **100%** | **698** | **100%** | **2,170** | **100%** |

# Supplementary Table 3.Treatment Arm and Disease Characteristic of Patients in this Substudy Compared to the CHHiP Trial as a Whole

| **Characteristic** | **This Study** | | **Whole XXXXXX Trial** | |
| --- | --- | --- | --- | --- |
|  | **No.** | **%** | **No.** | **%** |
| **Age** | **69 years** | **44-85 (range)** | **69 years** | **44-85 (range)** |
| **Arm (Intent to Treat)** |  |  |  |  |
| 57 Gy / 19 Fractions | 802 | 34% | 1077 | 33% |
| 60 Gy / 20 Fractions | 791 | 34% | 1074 | 33% |
| 74 Gy / 37 Fractions | 757 | 32% | 1065 | 33% |
| **Regimen Received** |  |  |  |  |
| 57 Gy / 19 Fractions | 799 | 34% | N/A | N/A |
| 60 Gy / 20 Fractions | 789 | 34% | N/A | N/A |
| 74 Gy / 37 Fractions | 762 | 32% | N/A | N/A |
| **NCCN Risk Group** |  |  |  |  |
| Low risk | 325 | 14% | 484 | 15% |
| Intermediate risk | 1,750 | 75% | 2347 | 73% |
| High risk | 275 | 12% | 385 | 12% |
| **Gleason score** |  |  |  |  |
| ≤6 | 792 | 34% | 1122 | 35% |
| 7 | 1,488 | 63% | 1995 | 62% |
| 8 | 70 | 3% | 99 | 3% |
| **Clinical T Stage** |  |  |  |  |
| T1 | 887 | 38% | 1170 | 36% |
| T2 | 1,275 | 54% | 1766 | 55% |
| T3 | 186 | 8% | 277 | 9% |
| Missing | 2 | <1% | 3 | <1% |
| **Pre-Treatment PSA** |  |  |  |  |
| <10 ng/mL | 1,141 | 49% | 1567 | 49% |
| 10-20 ng/mL | 1,072 | 46% | 1415 | 44% |
| ≥20 ng/mL | 137 | 6% | 208 | 6% |
| Missing | 0 | 0% | 26 | <1% |
| **Total** | **2350** | **100%** | **3216** | **100%** |

# Supplementary Figure 1. Example of Whole Rectum Contour Review Causing Longer Rectal Length But Reduced Rectal Volume

An example patient to demonstrate how rectal contour review can result in an increased rectal length while also a reduced volume. The blue contour is the original whole rectum and the red contour is reviewed rectum. The inferior limit has been extended to reach the anus. The superior border has been reduced to where the rectum turns anteriorly, removing some sigmoid colon.


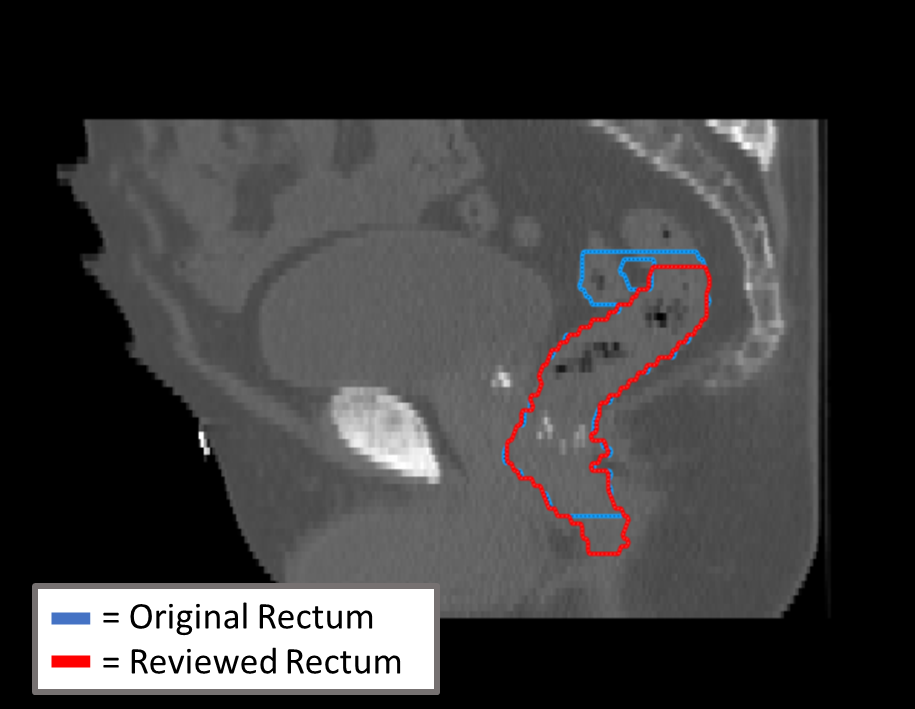


# Supplementary Figure 2. Dice Similarity Coefficients Between Original and Reviewed Rectum for Whole and PTV-Truncated Rectum.

The DSC score is calculated between the original and reviewed rectum for whole rectum and two truncations (PTV±2cm and PTV±0cm).The DSC agreement increases with tighter truncation to the PTV, which might be expected given the potential for inter-observer variation in the superior and inferior aspects of rectum.


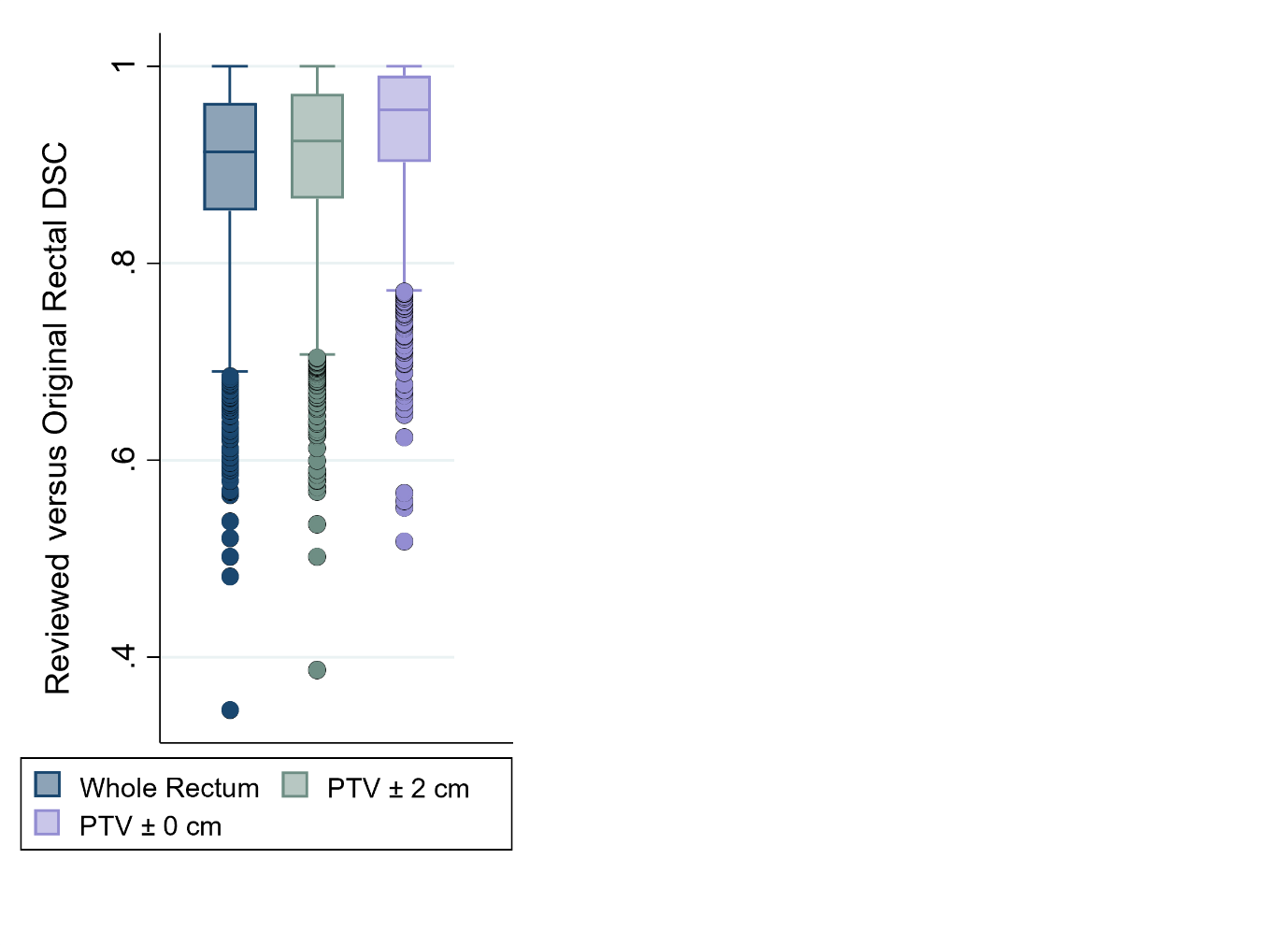


# Supplementary Figure 3. Volumes at each dose-level of interest for the original whole rectum, by trial arm.

The median dose-levels for the 60Gy and 57Gy arms are EQD2-adjusted (α/β ratio = 3 Gy). The top panel shows relative volumes, while the bottom panel shows absolute volumes. Inter-quartile ranges are shown as vertical bars.

# Supplementary Table 4. Volumes at each dose-level of interest for the original whole rectum, by trial arm.

A summary table giving the mean and standard deviation volume at each dose level of interest , for each rectal definition examined in the manuscript: Original whole rectum with relative volumes; Reviewed whole rectum with relative volumes; truncated rectum with relative volumes (PTV+2cm, PTV+0cm); original whole rectum absolute volumes.

| **Dose Level of Interest** | **Original** | | **Reviewed** | | **PTV+2cm** | | **PTV+0cm** | | **Absolute**  **Volumes** | |
| --- | --- | --- | --- | --- | --- | --- | --- | --- | --- | --- |
|  | **Mean**  **(%)** | **SD**  **(%)** | **Mean**  **(%)** | **SD**  **(%)** | **Mean**  **(%)** | **SD**  **(%)** | **Mean**  **(%)** | **SD**  **(%)** | **Mean**  **(cc)** | **SD**  **(cc)** |
| **V30*** | 68.1 | 15.4 | 67.0 | 14.7 | 69.4 | 14.7 | 81.6 | 14.6 | 48.2 | 18.7 |
| **V40*** | 49.0 | 14.8 | 48.4 | 14.4 | 50.0 | 14.6 | 59.6 | 15.9 | 34.7 | 15.4 |
| **V50*** | 36.3 | 12.4 | 35.9 | 12.3 | 37.1 | 12.3 | 44.6 | 14.0 | 25.9 | 12.5 |
| **V60*** | 21.6 | 9.5 | 21.3 | 9.4 | 22.1 | 9.6 | 26.8 | 11.3 | 15.4 | 8.7 |
| **V65*** | 11.7 | 6.6 | 11.5 | 6.6 | 12.0 | 6.7 | 14.6 | 8.1 | 8.3 | 5.6 |
| **V70*** | 3.4 | 3.8 | 3.3 | 3.8 | 3.5 | 3.9 | 4.2 | 4.7 | 2.4 | 3.0 |
| **V74*** | 0.1 | 0.5 | 0.1 | 0.5 | 0.1 | 0.5 | 0.1 | 0.5 | 0.1 | 0.4 |

# Supplementary Figure 4. Volumes at Each Dose Level of Interest

Comparison of median volumes between original and reviewed rectal contours, at each dose level of interest, for Whole Rectum and two truncated rectums (PTV±2cm and PTV±0cm). Panel A shows relative volumes and panel B shows absolute volumes. Significant differences (multiple comparison adjusted) at each dose level are marked with a cross. Inter-quartile ranges are shown as vertical bars.

Panel A – Relative Volumes


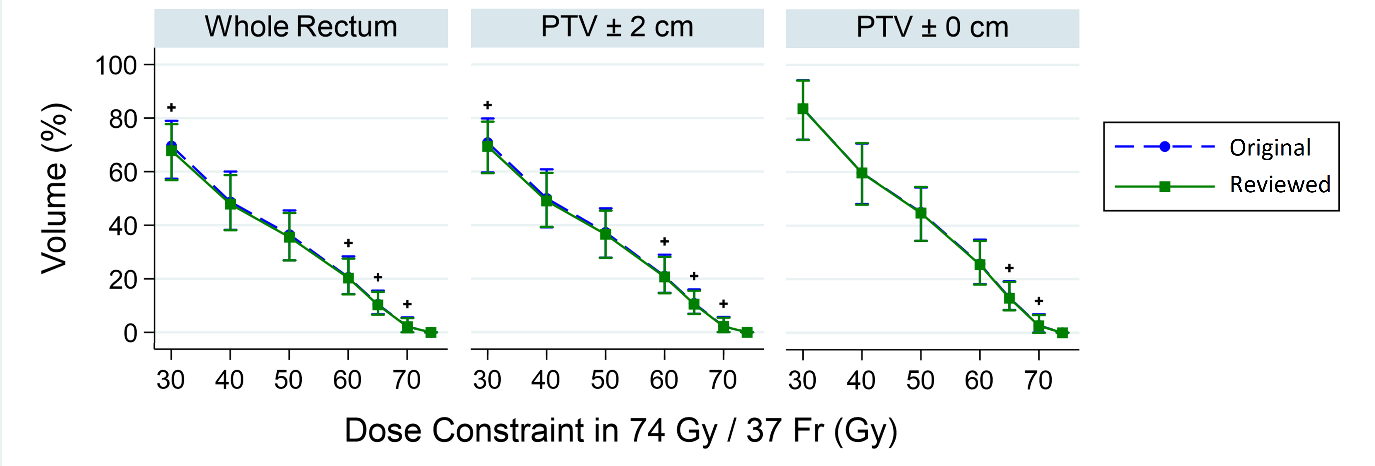


Panel B – Absolute Volumes


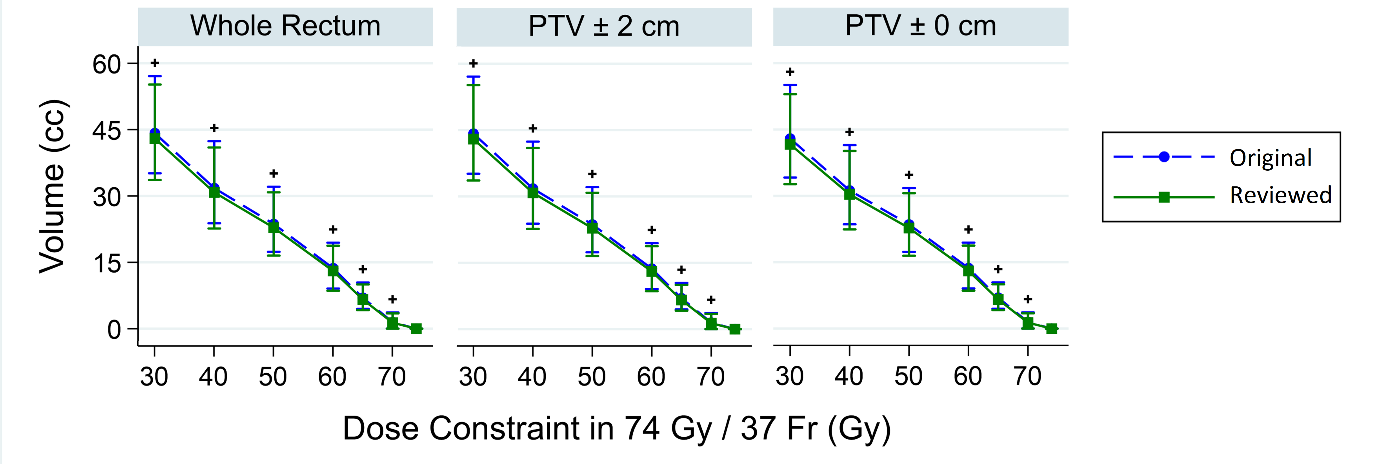


# Supplementary Table 5. Rectal Toxicity Prediction: Whole Rectum vs PTV±2cm

For original whole rectum contours and PTV±2cm, logistic models fitted to relative (%) DVH dose levels: V30*, V40*, V50*, V60*, V65*, V70* and V74* for each endpoint. The sensitivity, specificity, positive predictive value and negative predictive value are estimated by 632 method. The predictive ability (AUC) is compared between whole rectum and PTV±2cm contours, with no difference by DeLong comparison, with no significant differences seen.

Abbreviations: AUC = Area Under Curve; 95% CI = 95% confidence interval; NPV = negative predictive value; PPV = positive predictive value; Sens = sensitivity; Spec = specificity.

| **Endpoint** | **n** | **Whole Rectum** | | | | | | **Truncated Rectum: PTV**±**2cm** | | | | | | **AUC: Whole Rectum vs PTV**±**2cm**  **p-value** |
| --- | --- | --- | --- | --- | --- | --- | --- | --- | --- | --- | --- | --- | --- | --- |
|  |  | **AUC** | **AUC**  **95% CI** | **Sens**  **632** | **Spec**  **632** | **PPV**  **632** | **NPV**  **632** | **AUC** | **AUC**  **95% CI** | **Sens**  **632** | **Spec**  **632** | **PPV**  **632** | **NPV**  **632** |  |
| **Frequency G1+** | 1986 | **0.57** | 0.54–0.60 | 0.46 | 0.66 | 0.45 | 0.67 | **0.57** | 0.55–0.60 | 0.45 | 0.66 | 0.45 | 0.66 | 0.2361 |
| **Frequency G2+** | 1982 | **0.60** | 0.56–0.64 | 0.61 | 0.56 | 0.18 | 0.90 | **0.60** | 0.56–0.64 | 0.61 | 0.56 | 0.18 | 0.90 | 0.9898 |
| **Bleeding G1+** | 1969 | **0.60** | 0.57–0.63 | 0.42 | 0.73 | 0.44 | 0.72 | **0.60** | 0.58–0.63 | 0.49 | 0.66 | 0.41 | 0.73 | 0.0512 |
| **Bleeding G2+** | 1967 | **0.60** | 0.57–0.64 | 0.48 | 0.66 | 0.19 | 0.88 | **0.60** | 0.57–0.64 | 0.45 | 0.69 | 0.20 | 0.88 | 0.9854 |
| **Proctitis G1+** | 2105 | **0.58** | 0.56–0.61 | 0.55 | 0.55 | 0.40 | 0.70 | **0.58** | 0.56–0.61 | 0.54 | 0.56 | 0.41 | 0.70 | 0.7567 |
| **Proctitis G2+** | 2104 | **0.57** | 0.54–0.61 | 0.66 | 0.43 | 0.12 | 0.92 | **0.57** | 0.54–0.61 | 0.62 | 0.47 | 0.12 | 0.91 | 0.9585 |
| **Sphincter Control G1+** | 2154 | **0.61** | 0.57–0.65 | 0.67 | 0.48 | 0.14 | 0.92 | **0.61** | 0.57–0.65 | 0.60 | 0.55 | 0.14 | 0.92 | 0.1463 |
| **Stricture/Ulcer G1+** | 2161 | **0.65** | 0.59–0.71 | 0.65 | 0.57 | 0.05 | 0.98 | **0.65** | 0.59–0.71 | 0.68 | 0.54 | 0.05 | 0.98 | 0.5129 |

# Supplementary Table 6. Rectal Toxicity Prediction: Whole Rectum vs PTV±0cm

For original whole rectum contours and PTV±0cm, logistic models fitted to relative (%) DVH dose levels: V30*, V40*, V50*, V60*, V65*, V70* and V74* for each endpoint. The sensitivity, specificity, positive predictive value and negative predictive value are estimated by 632 method. The predictive ability (AUC) is compared between whole rectum and PTV±0cm contours, with no difference by DeLong comparison, with no significant differences seen.

Abbreviations: AUC = Area Under Curve; 95% CI = 95% confidence interval; NPV = negative predictive value; PPV = positive predictive value; Sens = sensitivity; Spec = specificity.

| **Endpoint** | **n** | **Whole Rectum** | | | | | | **Truncated Rectum: PTV**±**0cm** | | | | | | **AUC: Whole**  **Rectum vs**  **PTV**±**0cm**  **p-value** |
| --- | --- | --- | --- | --- | --- | --- | --- | --- | --- | --- | --- | --- | --- | --- |
|  |  | **AUC** | **AUC**  **95% CI** | **Sens**  **632** | **Spec**  **632** | **PPV**  **632** | **NPV**  **632** | **AUC** | **AUC**  **95% CI** | **Sens**  **632** | **Spec**  **632** | **PPV**  **632** | **NPV**  **632** |  |
| **Frequency G1+** | 1986 | **0.57** | 0.54–0.60 | 0.46 | 0.66 | 0.45 | 0.67 | **0.57** | 0.55–0.60 | 0.54 | 0.57 | 0.43 | 0.67 | 0.3831 |
| **Frequency G2+** | 1982 | **0.60** | 0.56–0.64 | 0.61 | 0.56 | 0.18 | 0.90 | **0.59** | 0.55–0.63 | 0.56 | 0.58 | 0.18 | 0.90 | 0.3569 |
| **Bleeding G1+** | 1969 | **0.60** | 0.57–0.63 | 0.42 | 0.73 | 0.44 | 0.72 | **0.60** | 0.58–0.63 | 0.49 | 0.67 | 0.42 | 0.73 | 0.4947 |
| **Bleeding G2+** | 1967 | **0.60** | 0.57–0.64 | 0.48 | 0.66 | 0.19 | 0.88 | **0.60** | 0.57–0.64 | 0.51 | 0.63 | 0.19 | 0.89 | 0.6724 |
| **Proctitis G1+** | 2105 | **0.58** | 0.56–0.61 | 0.55 | 0.55 | 0.40 | 0.70 | **0.58** | 0.56–0.61 | 0.53 | 0.57 | 0.40 | 0.69 | 0.6827 |
| **Proctitis G2+** | 2104 | **0.57** | 0.54–0.61 | 0.66 | 0.43 | 0.12 | 0.92 | **0.57** | 0.53–0.61 | 0.46 | 0.62 | 0.13 | 0.91 | 0.3075 |
| **Sphincter Control G1+** | 2154 | **0.61** | 0.57–0.65 | 0.67 | 0.48 | 0.14 | 0.92 | **0.61** | 0.58–0.65 | 0.56 | 0.60 | 0.15 | 0.92 | 0.2668 |
| **Stricture/Ulcer G1+** | 2161 | **0.65** | 0.59–0.71 | 0.65 | 0.57 | 0.05 | 0.98 | **0.66** | 0.60–0.71 | 0.70 | 0.53 | 0.05 | 0.98 | 0.9836 |
